# Determinants of Food Waste in Cluj-Napoca (Romania): A Community-Based System Dynamics Approach

**DOI:** 10.3390/ijerph20032140

**Published:** 2023-01-24

**Authors:** Bianca Cezara Archip, Ioan Banatean-Dunea, Dacinia Crina Petrescu, Ruxandra Malina Petrescu-Mag

**Affiliations:** 1 Faculty of Environmental Science and Engineering, Babeș-Bolyai University, 30 Fantanele Street, 400294 Cluj-Napoca, Romania; 2 Biology and Plant Protection Department, Faculty of Agriculture, University of Life Sciences “King Mihai I” from Timisoara, 119 Calea Aradului, 300645 Timisoara, Romania; 3 Department of Hospitality Services, Faculty of Business, Babeș-Bolyai University, 7 Horea Street, 400174 Cluj-Napoca, Romania; 4 Department of Economy and Rural Development, Faculty of Gembloux Agro-Bio Tech, University of Liège, Passage des Déportés 2, 5030 Gembloux, Belgium; 5 Doctoral School “International Relations and Security Studies”, Babes-Bolyai University, 1 M. Kogalniceanu Street, 400084 Cluj-Napoca, Romania

**Keywords:** stakeholders, perceptions, system thinking, waste, sustainable development

## Abstract

This study identifies the most relevant causes of food waste according to the perceptions of key stakeholders in Cluj-Napoca, Romania. Community-Based System Dynamics (CBSD), a qualitative approach, was used to reveal the determinants of food waste. CBSD was intended to encourage the system thinking of participants in the field of food waste. Consequently, CBSD helped us map and visualize the role of each identified cause in the system and the nature of their interactions. For the present study, four categories of stakeholders were involved: consumers, public administration, food waste business, and the NGO sector involved in food waste reduction. The result of each modeling session was a loop diagram of the main food waste determinants. A common perception reflected within each stakeholder group was that food waste could be minimized through upstream actions. The participants highlighted pro-environmental knowledge, awareness, and values as the prerequisites for fighting food waste. It was found that the lack of education and awareness of food waste directly impacted food waste generation. In addition, the role of education was underlined by participants as a contributor to changing individual and household practices, such as overbuying. The lack of connection between consumers and the food production process, coupled with consumerist practices and the rejection of ‘ugly food’, contributed to the decrease in the overall value people attributed to food. Governmental intervention, through legislation, was indicated by the CBSD participants as being key to increasing societal awareness and shaping the behavior of food chain actors. We concluded that food waste is a ‘wicked problem’ and the interlocking of the economic, social, political, and environmental spheres and the multitude of stakeholders’ interests, values, and perceptions should be considered in designing sustainable solutions to combat food waste. Finally, this research testifies to the importance of engaging with diverse panels of stakeholders who, through the multitude of opinions and perspectives on the causes of food waste, can further create knowledge about the most appropriate ways to combat the food waste phenomenon.

## 1. Introduction

In recent years, the phenomenon of food waste has increased the attention of researchers, policymakers, environmentalists, and social activists [1,2,3] as one of the urgent cross-cutting issues of the environmental, economic, and social sectors. The complexity of food waste requires the consideration of the principle of sustainability in problem-solving strategies [4,5]. Combating food waste is included in the ‘UN 2030 Agenda for Sustainable Development’. According to Goal 12, Responsible consumption and production, by the end of the decade, global food waste must be halved at retail and consumer levels and food loss must be reduced [6]. Reducing food waste would also be relevant for reaching other goals of the Agenda: 1. No poverty, 2. Zero Hunger, 8. Decent work and economic growth, 13. Climate action, or 15. Life on land [6]. However, some of the food waste combat desiderata were put on standby in the last two years due to the COVID-19 crisis. “Stay-at-home” and “remote-working” strategies to prevent the spread of COVID-19 made many of us consume more than what was needed [7,8,9]. We witnessed dramatic changes in food consumption habits, due to the COVID-19 lifestyle and psychological stress, that influenced the generation of food waste along the supply chain [10,11].

Directive 2018/851 on waste highlights the need for better waste management at the EU level, the rational use of natural resources, promoting a circular economic model, and improving the energy sector [12]. Section (31) of the preamble of the directive provides guidelines for preventing and reducing food waste, aligned with the 2030 Agenda. The EU aims to reduce food waste by 30% before 2025 and 50% by 2030. These goals require the member states to take prevention and reduction measures (awareness campaigns, facilitate knowledge exchange, and food waste reporting). Additionally, the directive draws attention to the responsibility of member states to develop a favorable framework for collecting and redistributing unsold food and increasing awareness among consumers about the distinction between ‘use-by’ and ‘best-before’ dates [12]. At the national level, the Romanian legislation, Article 1 (2) of Law No. 217/2016 for the reduction of food waste [13], defines food waste as “the situation in which food leaves the circuit of human consumption due to degradation and is destroyed, according to the legislation in force”.

Identifying the leading causes of food waste is the ground zero to develop effective solutions to reduce its impact. However, this task is rather challenging, as economic, social, and political factors are intertwined in maintaining an economic model focused on constant growth and competition, which often fails to consider the natural environment and our pressure on it. Researchers and activists highlight the need to address social and environmental concerns within capitalist system thinking if we aim to down-scale prevalent problems such as inequality, poverty, climate change, pollution, or the depletion of natural resources [14,15]. The distribution of the economic benefits of capitalism is characterized by a high degree of inequality, both between and within states [16].

This study identifies the most relevant causes of food waste by revealing the perceptions of key stakeholders in the food waste sector, based in Cluj-Napoca, Romania. Engaging with relevant local actors in the food waste area offers valuable information on their perception of this phenomenon and informs the readers about the specificities of food waste causes in the Romanian context. The study provides a bottom-up perspective of food waste mitigation and can fill in some of the literature gaps on food waste in the cases of Romania and, in particular, Cluj-Napoca. We consider that the city of Cluj-Napoca represents a suitable case study for analyzing the causes of food waste, being one of the most populous cities in Romania and under continuous development [17]. These aspects justify the stringent need for effective waste management and pollution mitigation strategies [18,19]. Moreover, the municipality has attempted to become an example of innovation, good governance, and civil participation [17,20], aspects that would facilitate the implementation of future measures against food waste. For the purpose of the current study, we invited members of the local public administration, the business sector, the non-governmental sector, and consumers. As described in the Methodology section, we carried out thematic workshops with each of the four groups.

The Community-Based System Dynamics (CBSD) method is used to better understand the complex issues faced by a community. CBSD is a participatory research method with a relevant educational dimension based on the input of members of a specific community. It helps to map out and visualize the role that a particular problem has within the system of factors with which it interacts, as well as the nature of these interactions. Thematic modeling sessions about food waste causes were intended to increase the awareness and understanding of the problem, thus facilitating the identification of possible solutions to fight food waste [21,22].

On the topic of food waste, as well as the direct causes of food waste, no other study has used the qualitative approach of system thinking to reveal the stakeholders’ perceptions about the causes of the problem. We offer a more comprehensive understanding of the determinants of food waste compared to other studies, including a diverse panel of stakeholders. More precisely, other participatory approaches focused on determining the direct drivers of food waste, mainly from the perspective of the producer or the consumer (as per, [23,24,25,26,27]). Furthermore, this approach has the merit of highlighting the similarities and differences of perceptions between the actors, while providing a safe space in which each group can freely express their views and opinions. The findings can be valued as a ‘validation test’ for the fitness of current and future strategies aiming to strengthen the efforts to reduce food waste. The level of engagement of stakeholders in reducing food waste can be predicted based on their perception of the problem. Qualitative research can offer information on areas where action is needed most to reduce and combat food waste and predict the success of such measures as stakeholders are responsible for their development, dissemination, or implementation. Therefore, the objective of the study is to identify the causes of food waste in Cluj-Napoca using a CBSD approach.

The rest of the paper is organized as follows. The section ‘From food-to-food waste in Romania’ describes the socio-economic conditions and the role of agriculture in Romania, followed by data regarding food waste trends and the waste’s impact. This section ends with a brief analysis of the legislation on food waste in Romania. The Methodology section includes a short presentation of the research area of Cluj, a description of the stakeholders included in the study, and the way the modeling sessions/workshops have been organized. The Results are presented in Section 4, reporting the causes of food waste identified during the workshops, the loop diagram, and the main findings. The analysis and interpretation of the results follow. The final remarks are included in the last section.

## 2. From Food-to-Food Waste in Romania

### 2.1. The Romanian Food Sector and the Overall Socio-Economic Context

Poverty remains a socio-economic challenge in Romania, which is expected to aggravate due to the current global economic crisis and the post-pandemic context. In 2021, 34.4% of Romanians were at risk of poverty or social exclusion, ranking the worst among the EU states [28]. In the same year, Romanians spend more than a quarter of their income on food, the highest percentage among the EU states [29].

Agriculture is one of the key economic areas in Romania. It is estimated that in 2017, more than half (56%) of the total territory of Romania (23.84 million ha) represented agricultural land [30,31]. However, working in the agricultural sector has become less appealing due to low wages, especially for young people. The number of people working in the agricultural field has decreased from 28% in 2008 to 20% in 2020 [32].

Reducing food waste could improve the standard of living of many households in the country and the use of resources in agriculture. Although it can be challenging to measure the increase in turnover that farms would have if their products did not end up as waste, the opportunity cost of growing crops and animals that generate no income (because these crops end up as waste and are not sold) is substantial. Land area, water, fertilizers, food, medicine, machinery, and human resources invested in food that is wasted could be used for other purposes.

### 2.2. Extent, Intensity, and Consequences of Food Waste

Providing precise and reliable data on the overall extent of food waste is rather difficult due to inconsistencies in defining the problem, the diversity of actors, and the general complexity of the food chain (production, processing, transportation, retailing, usage, and disposal) [33,34]. For Romania, the lack of official food waste data is an additional challenge. Although the food waste data from other European countries are present in different EU statistics, Romania is not part of them [35].

At the global level, the World Food Programme (WFP) estimates that approximately 1/3 of the food produced for human consumption is wasted, which is worth around USD 1 trillion every year [36]. In 2019, 931 million tons of food waste were produced globally, with households generating 61% of it [37]. All this food could feed 2 billion people, “more than twice the number of undernourished people across the globe” [36].

According to the first large-scale analysis of the EUs food waste [38], 88 million tons (or 173 kg per person) of food were lost or wasted annually, costing around EUR 143 billion and accounting for 20% of the production of the economic block. However, new data were collected using the common methodology of the EU for monitoring food waste [39]. Eurostat estimates that 57 million tons of food were wasted (127 kg per person) in the EU in 2020, worth around EUR 130 billion [40]. Additionally, 10% of the food that reached EU consumers (households, food services, and retail) became waste. Households were responsible for 55% of the waste, followed by processing and manufacturing (18%), production (11%), restaurants/food services (9%), and retail (7%) [40]. Preventing food waste could alleviate the suffering of the 36.2 million people in the EU who “cannot afford a quality meal every second day” [41].

Studies revealed that, in Romania, more than 2.2 million tons of food are wasted annually, or approximately 2.5% of the total food waste produced at the Union level [42,43]. A study [44] that analyzed the food waste trends in Romania, the Republic of Moldova, and Macedonia concluded that 83% of the Romanian participants threw away food. At the same time, fruits, vegetables, and bread were the top three categories of foods that turned into waste at the household level. As mentioned previously, the 2020 analysis of the food waste produced in the EU states does not include any data on the situation in Romania [40]. However, the Food Waste Index Reports of UNEP (2021) show that Romanian households were responsible for 1.3 million tons of food waste (70 kg per inhabitant). The confidence in the accuracy of the data is ‘very low’ [37].

Environmental concerns, such as climate change and pollution, have also increased the relevance of tackling food waste [45,46]. The extent of the problem at the global level is rather severe and “if food loss and waste were a country, it would be the third biggest source of greenhouse gas emissions”, responsible for 8–10% of total emissions [37]. In Europe, agriculture accounts for approximately 10% of greenhouse gas (GHG) emissions [47]. Burning fossil fuels releases GHG emissions during all stages of the food’s lifecycle, from production to food processing, transportation, storage, and cooking. The food waste phenomenon adds an additional dimension to the environmental burden of the food system as decomposing unconsumed food produces relevant amounts of methane, a highly potent GHG [48,49,50]. If we consider all the stages during which GHGs are emitted, production is the highest contributor, accounting for around 73% of the impact. Thus, proactive approaches are highly needed and better waste management (e.g., composting) would only reduce 6% of the global warming potential of food waste [49].

However, the negative environmental impact of agriculture and, subsequently, food waste goes well beyond the concerns regarding GHG emissions and climate change. Land, freshwater, and fossil energy are needed for agricultural production and animal farming. At the global level, 23% of the total cropland area and 24% of the water for agriculture is used to produce food that will end up as waste [51], adding to the burden on our food systems.

In addition, pesticides and fertilizers are well-known sources of water and soil pollution [52]; food waste accounts for 23% of the fertilizers used globally [51]. Moreover, 12% of the diffuse nitrogen water pollution from the EUs agriculture can be attributed to food waste, increasing the eutrophication of water bodies and posing serious environmental consequences for the affected ecosystems [53]. Although measures are taken to address all these issues, being more aware of our food waste represents an opportunity to reduce the environmental impact of agriculture [54] and to better adapt our food system to future growing demands [55].

### 2.3. Addressing Food Waste in Romania

As part of the EU, Romania took some steps to address the issue of food waste. An example is Law No. 217/2016 on the reduction of food waste [13], which stipulates that reducing food waste is a relevant objective for all economic operators in the agri-food sector. Operators must take various measures to prevent food waste, such as discounting or donating food products that are about to reach the ‘sell-by’ date. Law No. 217/2016 has the merits of defining and acknowledging the problem of food waste and presenting the hierarchy of solutions for combating it. However, the law has been perceived as rather strict and rigid, especially in facilitating food donation. Currently, economic operators can donate nonperishable foods only during the last ten days before the ‘sell-by’ date of a product. Perishable foods (unpasteurized vegetable and fruit juices, precut vegetables and fruits, and sprouted seeds) and animal-origin products are not allowed to be donated [13,56].

This limitation is problematic when retailers and distributors become aware of their overstock, sometimes long before the ten-day framework. Since the law does not allow food donations before the last ten days of its shelf life, economic operators have no choice but to either provide storage for that stock until it can be donated or discard it to waste management companies. Both options are disadvantageous. Offering storage for overstocks occupies valuable space and increases the use of energy (e.g., light, cooling systems) for products that will bring no revenue. At the same time, discarding perfectly good products goes against the commitments to reducing food waste.

Another legislative project on reducing food waste passed the Romanian Senate in October 2022. The project proposes some relevant measures, such as developing a national online platform to report food waste data, developing a national strategy for food waste prevention and reduction, increasing responsibilities for public authorities and economic operators, and simplifying the food donation system [42]. According to the Romanian National Institute of Statistics, 34 receiving operators, such as food banks, are working to reduce food waste in Romania, 20 of them joining the efforts in 2021 [31].

## 3. Materials and Methods

Analyzing the food waste phenomenon in the case of Cluj-Napoca, Romania can help develop an in-depth view of the problem, its causes and consequences, and its specificities within the local community [57,58]. While the findings may not be fully applicable to an international context because of the qualitative approach [59], they can fill in the missing data on the food waste problem in Romania and highlight the need for further qualitative and quantitative investigations. Exploring the topic through this method provides the backbone for an interpretation and analysis of the stakeholders’ perceptions regarding the food waste causes. Moreover, a case study conceptualization and analysis helps to reveal the nexus between the environmental and social dimensions of food waste as a cross-cutting issue [60].

The analysis of a complex and multi-causal problem, such as food waste, requires an understanding of the factors that influence it and the relations between them. The system dynamics approach focuses on the simulation and modeling of the causal relationships that are specific to a problem and its context [22,61]. Group modeling is one of the methods used for system dynamics, which can ensure that relevant individuals (experts, practitioners, stakeholders, and members of a community) are directly involved [21].

CBSD proposes the same type of analysis, designed to encourage system thinking through modeling, by involving community members in the process [21]. CBSD is a qualitative approach in which the first-hand experience of community members represents the pillars of conceptualization during the modeling sessions. It provides a valuable setting for understanding and analyzing the problem, facilitates exchanging viewpoints, and finds solutions. Moreover, it creates a harmonized perception of the topic, which can be crucial for the future success of strategies and policies developed in the field [62]. For this study, we analyzed the causes of food waste with the help of four categories of food stakeholders from the local community of Cluj-Napoca. These are consumers, public administration, food waste businesses, and the NGO sector (acting in food waste reduction).

### 3.1. Study Area: Cluj County

Cluj County is located in the northwest of Romania and it is the center of the historical region of Transylvania. The county’s surface is 6674 km^2^ with a population of 71.1630 inhabitants [31]. The county municipality, the city of Cluj-Napoca, is one of Romania’s most important university centers.

The county generates around 335.000 tons of solid municipal waste annually (481 kg/capita) [63]. Currently, this waste is stored in inappropriate spaces, especially in illegal landfills [63]. The most striking case was in Pata Rât (Cluj-Napoca), the illegal landfill that became home to around 1500 people evacuated from Cluj-Napoca after the year 2000 [64]. In 2018, the European Court of Justice ruled that Romania had breached ‘Directive 1999/31/EC’ [65] as it did not close its illegal landfills, including Pata Rât [66]. After intensive pressures from environmental and human rights lobby groups, the landfill was closed and the area rehabilitated in 2019 [67].

### 3.2. Selection of Stakeholders and Interviews Development

In the present study, four relevant categories of stakeholders were considered: consumers, public administration, NGO, and a start-up business involved in the reduction of food waste. Two NGOs (based in Cluj-Napoca) were involved because they are active in food collection and redistribution (food banks), awareness-raising, the creation of educational campaigns and materials regarding food waste. The start-up business refers to a company that develops technological solutions for reducing food waste in commercial kitchens, by providing means of monitoring, measuring, and reporting food waste. A “commercial kitchen” is a cooking area that has been designed for food production, mainly for the hospitality industry (for example, bars, restaurants, and hotels).

We considered that including the public and civil society sectors was important for obtaining a proper image of the perceptions of food waste in Cluj-Napoca. After identifying our interest groups and organizations, the facilitator of the group used social media platforms to reach out to potential participants in each category, presenting the research topic, its objectives, and the methods. Participation was voluntary and there were no incentives offered. Those who wanted to participate in the study expressed their informed consent after the preliminary discussions and at the beginning of the modeling sessions. Although the list of potential food waste stakeholders is rather extensive, we decided to focus on four of the most relevant groups, with an important impact on the development and implementation of strategies and policies. We consider that effective food waste solutions will depend on the partnership between public, private, and civil society. This study can be considered a preliminary step in this direction.

Five group modeling sessions were held (between September and November 2022) with people that expressed their interest in participating in the study: eight consumers (C), one representative of the local public administration (administration/A), four employees of a start-up focused on food waste reduction in commercial kitchens (business/B), and two separate sessions with two local NGO leaders (NGO/N1, N2). Although we acknowledge that including more members in some of the groups would have been beneficial, time and network limitations generated the structure of the sessions.

The script used for the modeling sessions followed a similar structure for all groups. Still, slight modifications were made on the spot depending on how the group members interacted and their interests in the topic. The time of each session was between 1 h and 1.5 h. The beginning of the session was dedicated to a brief presentation of the research topic, the research purpose, and the methodology used. All groups were informed about the other categories of stakeholders participating in the study. The general rules for the workshop were established: 1. participants were encouraged to speak freely (there are no ‘right’ or ‘wrong’ answers); 2. to respect the opinions of other participants and to express their disapproval in a polite manner; and 3. participants were informed that their identities would not be disclosed and that they could leave the workshop at any time. Due to time limitations and the fact that the members of each group knew each other in advance, we decided not to dedicate any time to group exercises or energizers.

At the beginning of the sessions, we introduced the participants to systemic thinking, using two examples of loop diagrams: one concerning population increase/decrease [68] and another concerning cardiovascular disease [69]. We informed the participants that a similar loop diagram will be developed as the outcome of the sessions. Using a projector, the operational definition of food waste was presented to the groups: “food waste is understood as the situation in which food leaves human consumption due to degradation and is destroyed” [13].

A brief discussion took place with the members of the business group regarding food waste exceptions. For example, they considered that food meant for human consumption that is given to animals or transformed into biomass energy should not be considered as waste. Additionally, a member of the NGO 2 group referred to the natural resources (e.g., land, water, and fertilizers) that are also lost whenever food waste occurs and a member of the administration group argued that the definition of food waste should focus on the waste that occurs from retailer to consumer and exclude production/processing losses.

After this part of the session, each member of the group was asked to list three causes of food waste that they considered to be the most relevant [70]. We considered it to be beneficial to increase the level of complexity during the following steps. For the groups with more participants (the consumer and business groups), we used the Mentimeter.com software to gather the answers, ensuring that each person was able to provide their input without being influenced by other participants. The facilitator used the software to distribute the open-ended question, to which the participants anonymously responded using their phones after connecting through a unique code. The software then compiled all the answers into a Word Cloud which was then projected on the screen. The verbally given answers or the results generated by the software in a Word Cloud were placed on a large sheet of paper or a whiteboard when it was available. The groups with multiple members were encouraged to observe when some variables (or similar variations of the variable) appeared more than once, showing that numerous people voted for them as significant causes of food waste.

The facilitator thematically grouped the variables listed at this point in each session. Interestingly, all the groups focused on the variables related to an individual/household, retailer, and HORECA food waste while excluding factors related to production/processing. The participants were then asked to add other variables on the board that are direct or indirect causes of food waste. In order to stimulate the discussion and help the groups to generate new ideas, the facilitator suggested how the participants could identify new variables, asking some additional questions such as ‘What leads to ‘consumerism’?’, ‘What is the influence of variable ‘x’ on other factors/variables?’. These questions encouraged the participants to consider new factors and reflect on the indirect causes of food waste [71]. The purpose was to build a comprehensive image of the causes of food waste to illustrate, at least in part, the complexity of the problem.

To create a safe space in which every participant could feel comfortable taking the floor, the facilitator proposed to do a round of ‘passing the pen’ from one individual to another. This ensured that everyone had the chance to speak. After a simplified/codified version of the idea was agreed upon with the participants, the facilitator added a new variable to the whiteboard. Next, the connections between that new variable and others were drawn. Nonetheless, whenever the discussion evolved organically, the facilitator did not interfere, and ‘passing the pen’ was resumed after the dialogue had stopped. Additionally, whenever the group had difficulties generating new ideas, the facilitator redirected them to elements from the board that had not been discussed so far. As the exercise progressed, we observed that the group participants became more comfortable adding variables and creating links between them. It was interesting that, right at the end of the brainstorming (when the last round of ‘passing the pen’ was announced), the business group started to identify some variables that, they suggested, could be relevant for all other variables (e.g., ‘education’ or ‘economic system’).

In the next part of the session, the facilitator explained to the groups how reinforcement and balance causal relationships work, using the ‘population increase/decrease’ example from the beginning of the exercise [68]. The participants were encouraged to focus on the links identified up to this point and assess how the variables influence each other. To simplify the process, the facilitator eliminated the negations attached to the variables whenever possible. The business group identified that some variables, for example ‘economic growth’ or ‘social media’, could be considered as both reinforcing (R) and balancing (B) for other variables (e.g., social media can raise food waste awareness and encourage a consumerist and wasteful lifestyle). The workshop ended after each group was asked to take a last look at the board and add anything missing. Finally, the facilitator thanked the members for their participation and informed them of the future steps of the study.

## 4. Results

### 4.1. Food Waste Causes That Emerged from the CBSD Approach

The variables discussed during the sessions can be seen in Table 1. Some of them have been standardized (common names) in the cases in which similar ideas, but with different names, were presented in multiple groups (e.g., ‘education’ appeared as a variable in all groups, although there were distinctions made between formal and informal education, or specific disciplines such as nutrition, critical thinking, and ecology). However, differences in the interpretation of the same variable within different groups are illustrated in Table 1.

### 4.2. Loop Diagram

**Figure 1:**
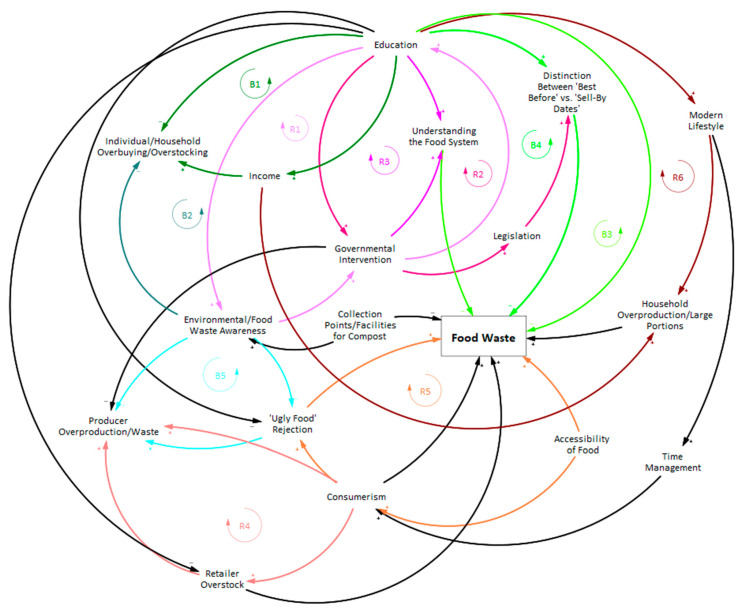
Food waste loop diagram, including the variables and their relations mentioned during multiple modeling sessions (each color highlights a distinct loop).

**Figure A1:**
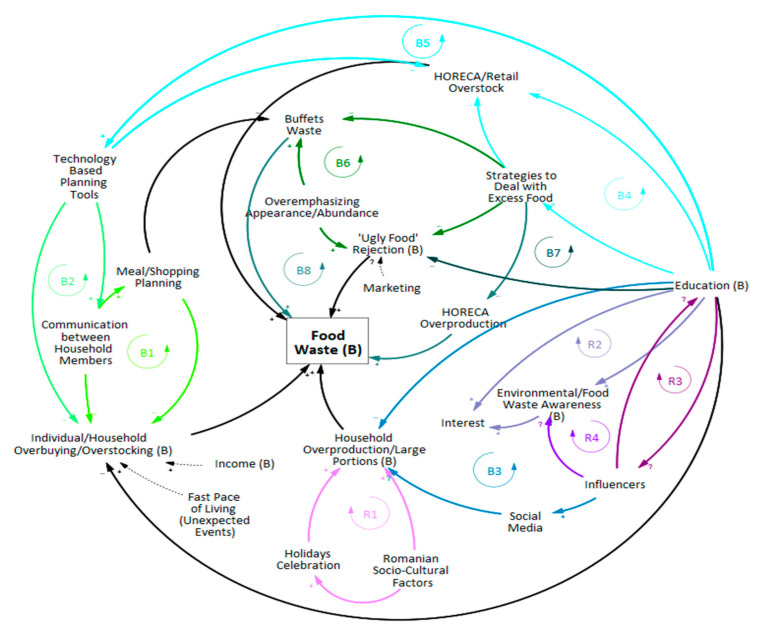
Food waste loop diagram elaborated during modeling session with the representatives of the business group (each color highlights a distinct loop).

**Figure A2:**
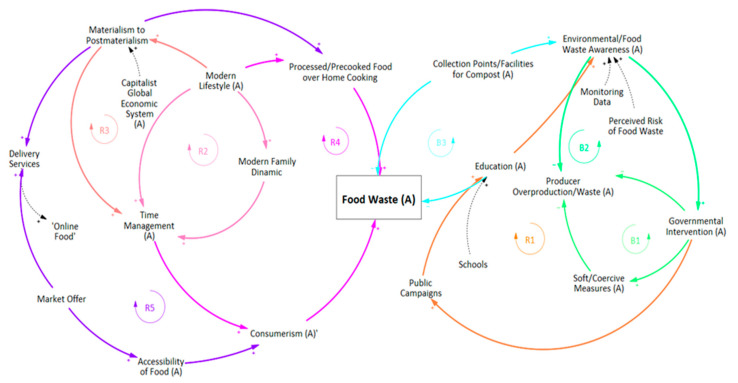
Food waste loop diagram elaborated during modeling session with the representative of the public administration (each color highlights a distinct loop).

**Figure A3:**
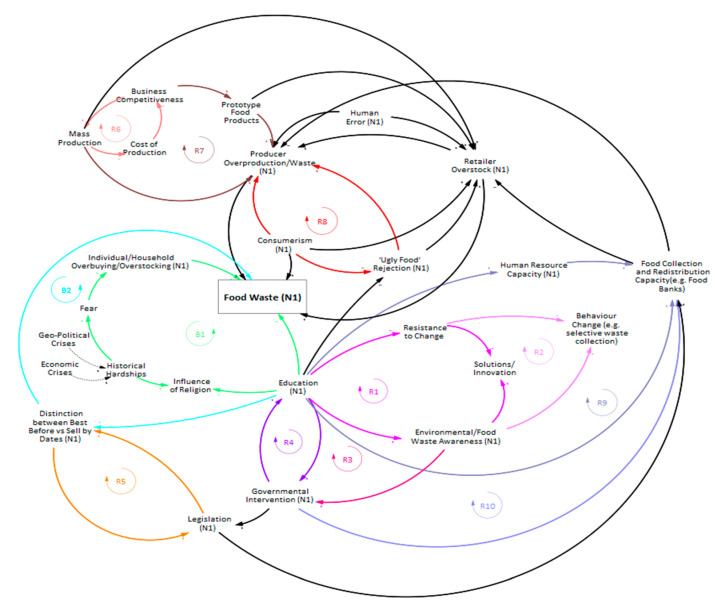
Food waste loop diagram elaborated during modeling session with the representative of NGO 1 (each color highlights a distinct loop).

**Figure A4:**
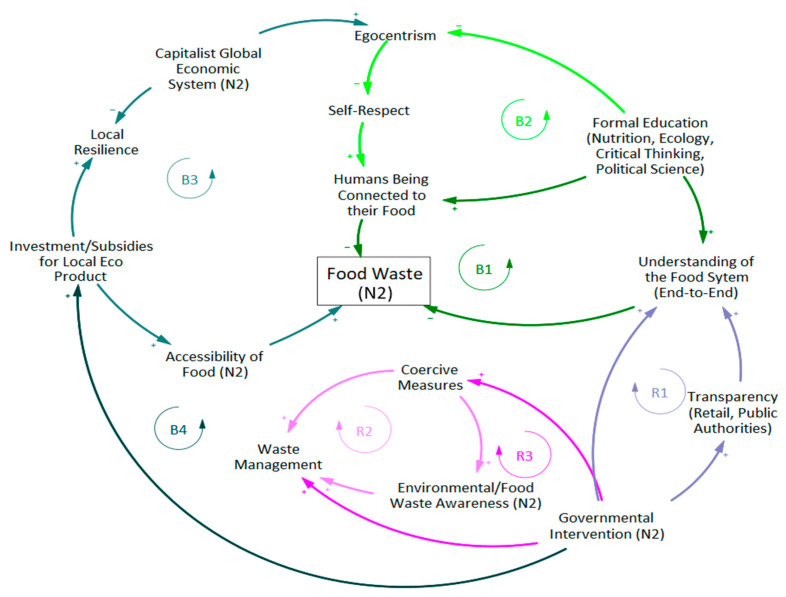
Food waste loop diagram elaborated during modeling session with the representative of NGO 2 (each color highlights a distinct loop).

**Figure A5:**
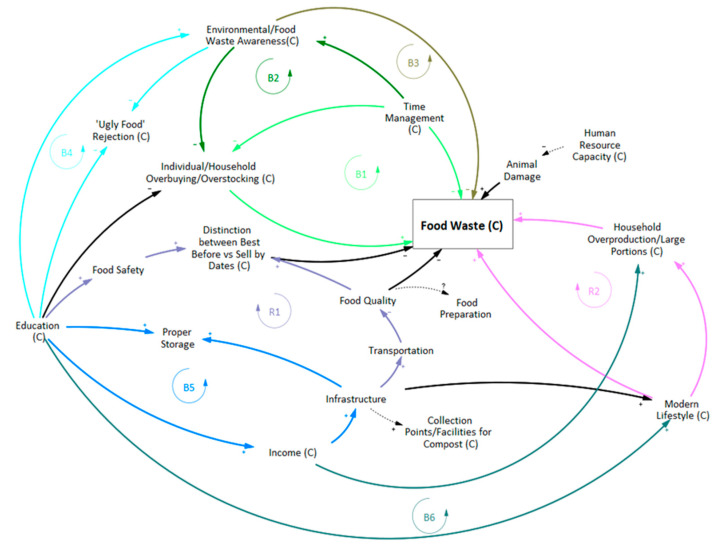
Food waste loop diagram elaborated during modeling session with the representatives of consumers (each color highlights a distinct loop).

The outcome of each modeling session was a loop diagram of the main food waste determinants. During the sessions, the causes of food waste and the relations between them were identified. The balancing and reinforcing loops were determined in the aftermath of the meetings. The diagrams for each stakeholder group are included in Appendix A (Figure A1, Figure A2, Figure A3, Figure A4 and Figure A5). The final loop diagram (Figure 1) includes the variables mentioned during more than one session. The relations between the variables were established based on the group modeling sessions. To better visualize each of the reinforcing and balancing loops of Figure 1, we included Table 2, which presents all the constitutive elements of the identified reinforcing and balancing loops.

## 5. Discussion

Stakeholders’ perceptions of the determinants of food waste were identified based on the modeling sessions that produced the loop diagrams. The results consist of a comprehensive list of factors which impact the generation of food waste and the diagrams highlight the interactions between them. Although many of the causes identified during our exercises are mentioned in the literature on the topic [9,23,70], our aim was to provide an aggregated image of the main causes of food waste and the actors involved. Moreover, it highlights the specificities of the Romanian context, derived from their historical, social, political, and economic background.

Some important variable categories can be derived from the answers provided by the groups during the modeling sessions. An important theme is related to the educational dimension. Multiple groups considered that a lack of education and awareness of food waste and the environment directly impacts the scaling of the problem. In the same category, we can include the awareness of different expiration dates or a proper (end-to-end) understanding of the food system. The need for better consumer awareness of the different expiration dates of food products has been identified in other studies and is included in the EUs policies [12,72]. Furthermore, consumers cannot properly assess the value of food due to a lack of understanding of the food system and its processes [70].

The evolution of the relationship between humans and food is another important cause of food waste at the individual level. The lack of a connection between people and their food, coupled with consumerist practices [73] and ‘ugly food’ rejection [74], marks the decrease in the overall value that people attribute to food. The rise in income and the widespread availability of food (quantity and variety), as a result of the capitalistic, competitive market, are some of the reasons for these behaviors. Consumerism, abundance, and low food prices diminish the incentives to reduce overbuying [70]. Additionally, this economic reality created a set-up where modern people can shift their interest in food. Since we can now buy precooked meals or use delivery services, spending time doing pleasant activities is considered more important.

While modern socio-economic factors impact consumer behavior, historical aspects are also relevant to the Romanian case. Periods of historical hardship experienced by society remain impactful on how people relate to food if we consider the tendency to overbuy, stock food, or prepare large meals. Changing behaviors can be difficult, especially if communities are resistant to change. Furthermore, the overall socio-economic context (e.g., economic crisis and post-pandemic realities) and consumer’s demand and expectations have an impact on the behavior of other relevant actors in the food chain (e.g., HORECA overproduction, retail overstock, and producer overproduction or buffet waste), which contribute to food waste [75,76,77].

Moving on, we can argue that the stakeholders’ perceptions illustrate the complexity of the food waste phenomenon. The groups identified various relevant actors: from individuals/households to public administration, retailers, HORECA, producers, and influencers. In addition, the groups could observe the relations between these actors. For example, governmental intervention can impact how producers and retailers operate, through legislation, coercive measures, or the responsible redistribution of financial resources toward local farmers. Another example is the interaction between individuals, retailers, and producers. The consumer’s demand for a wide variety of products that are easily accessible is a direct cause of ‘retailer overstock’. This factor has a further impact on ‘overproduction’. Considering the same actors, the rejection of ‘ugly food’ at the consumer level has a direct impact on the food waste produced by both retailers and producers. However, since 2009, there have been no legislative requirements regarding the appearance of food [78,79,80].

Economic, social, and political factors have been identified as relevant causes of food waste. Economic factors, such as ‘income’ or the ‘capitalist global economic system’ have an impact on the behavior of consumers (‘overbuying’/ ‘consumerism’) as well as on that of producers, retailers, and HORECA, who are in a constant competition on the market to match customers’ expectations.

Social factors, especially those related to the educational sphere (‘education’, ‘awareness’, and ‘understanding of the food system’), play a relevant role in the food waste system, with direct and indirect impacts on the behavior of all actors. An interesting observation lies in the dual relationship between the educational and the political variables: increasing public awareness regarding the food waste problem is a powerful incentive for government action. At the same time, public intervention, through campaigns and policies, can improve the understanding of food waste among citizens. In fact, many studies indicate that education and awareness campaigns can reduce food waste [81,82]. However, as signaled by the participants in our study, scaling up these efforts is necessary to achieve widespread behavioral and systemic changes.

Public authorities have the capacity/responsibility to signal the relevance of combating food waste and provide large-scale formal education focused on the food system and its environmental consequences. Furthermore, the development and implementation of legislative measures has direct consequences for other actors. The consumer and administration groups mentioned the need for ‘collecting points/facilities for compost’. Although such a measure does not directly impact food waste reduction and constitutes a reactive measure to the problem, it highlights the need for better waste management, which is very stringent in Romania. Composting has economic benefits and reduces the negative environmental impact of waste [83].

From a practical perspective, the study brought to the fore the views of various stakeholders in the food waste sector of Cluj-Napoca, which could inform policymaking to design bottom-up food waste reduction strategies and create bridges between experts, practitioners, policymakers, and citizens. The participation of public administration and start-up business representatives in the interviews can ease, for example, the identification of organizational weaknesses that may play a role in the occurrence of food waste. Feedback from all these stakeholder categories facilitates the development and implementation of measures to combat food waste [84], providing guidelines for strategic and cohesive planning, which considers the interaction between various food waste-related variables. Furthermore, regardless of the stakeholder category, the findings show that we should all be more aware and responsible for addressing food waste. Prevention must be prioritized amongst food waste management strategies.

In summary, this study illustrates the complexity of the food waste phenomenon and the need for a further elaboration of creative solutions that consider the diversity of the actors involved. It also highlights the importance of educational and informational campaigns and public administration interventions in increasing the level of awareness and reaction capacity of consumers, producers, retailers, and HORECA in combating food waste.

## 6. Conclusions

The current study focused on the main determinants of food waste, identified using the CBDS participatory approach. The CBSD was used to develop thematic modeling sessions with five groups (consumers, local public administration, the business sector, and non-governmental sector). The findings illustrated that food waste could be regarded as a ‘wicked problem’. Its complexity, implications on economic, social, political, and environmental spheres, the number of actors involved, and the multilevel dynamic between local, national, and international factors should be considered when developing food waste management solutions. Several theoretical contributions, managerial implications, and future research directions can be derived from the present study.

Considering the theoretical contribution of the study, we highlight that there are different evaluative tools for decision-making in waste management, for example, the environmental impact assessment, risk assessment, or system thinking. Although system dynamics was applied in different areas related to waste management (e.g., urban waste management, packaging waste management, hospital waste management, and food waste management), participatory system dynamics modelling, such as CBSD, is less present in the waste literature. The research highlighted the interactions between the causal variables that helped to create a clearer image on the dynamism of the food waste system. Thus, the existence of reinforcing loops and balancing feedback loops was outlined. The reinforcing loops amplified the dynamic system patterns of behavior showing that solutions to counteract food waste should mainly target their constituent variables. In other words, sustainable food waste solutions must attempt to “break” the vicious circle of reinforcing food waste factors.

From a managerial perspective, the reduction of food waste is beneficial at both the individual and the business levels. The findings revealed that the participants considered the lack of education and awareness of food waste as having direct amplifying impacts on food waste generation. Therefore, the strategies to combat food waste should support education and information campaigns that can mitigate food waste. For example, they can reveal the negative social, economic, and environmental impacts of egocentrism, overconsumption, or overstocking. Understanding the causes of food waste could stimulate prevention or minimization behaviors regarding food waste, both in private life and in companies. Another intervention point derived from identified food waste causes is the development of compost infrastructure that can reduce the environmental burden of landfilling food waste, while generating income and organic fertilizer. Therefore, the public administration should create a network of composting points that are nearby, user-friendly, and safe for community needs. Additionally, because participants named “ugly food” rejection as a food waste cause, retailers should find ways to increase their acceptance, for example, through discounting, while farmers could direct them to processors where appearance is not relevant.

The results should be considered in the context of several limitations. The small number of participants restricted the generation of a higher number of food waste causes. Including larger and more diverse stakeholder groups in further research can reflect the perspectives of all parties involved in the food system. For example, producers, retailers, and waste management companies can provide valuable information on the causes of food waste or the limitations each group has in addressing it. Moreover, carrying out common sessions with various stakeholder groups can be an opportunity to spark debate and ensure the flow of ideas between parties. Another possible improvement is a higher number of facilitators, responsible for mediating the sessions and elaborating the loop diagram. In this way, any possible personal bias can be minimized.

Finally, fragmented and ineffective decision-making, path-dependency, and the lack of proper communication and engagement between various actors and institutions in the field of waste management remain serious challenges in Romania. That is why engaging local communities and stakeholders in participatory research remains crucial to policymaking in the field of food waste.

## Figures and Tables

**Table 1:** List of variables discussed during the modeling sessions.

Food Waste Causes	Groups ^1^	Explanation Based on Participants’ Understanding
Accessibility of food	A, N2	Easy access to food resources (large variety, multiple retailers) can diminish the importance that a community attributes to food. A: reference to precooked meals.
Animal damage	C	Destruction of crops by wild animals.
Behavior change	N1	How individuals/communities can adapt how they act in a given context (a reference to selective waste collection).
Buffets waste	B	Food waste generated by buffets refers to both overproduction and waste that consumers generate by overfilling their plates.
Business competitiveness	N1	Having an advantage over competitors in the market, being preferred by consumers and obtaining good profits.
Capitalist global economic system	A, N2	World-wide economic system that promotes continuous growth by encouraging competition.
Coercive measures	A, N2	Obligations/restrictions imposed by the public authorities meant to correct a specific issue in a community.
Collection points/facilities for compost	A, C	Infrastructure needed to deal with biodegradable waste. A: as a sensitive issue in the urban area.
Communication between household members	B	Regarding food shopping, wanted meals, and distributions of tasks regarding buying/cooking. Can reduce instances of overbuying/overcooking.
Consumerism	A, N1	The constant increase in purchasing goods and services.
Cost of production	N1	Material, financial, and human resources used to produce a particular product.
Delivery services	A	Reference to retailers/HORECA.
Distinction between ‘best before’ vs. ‘sell-by dates’	N1, C	Understanding the difference between terms and their meaning, as reflected in legislation, public awareness, and behavior.
Economic crises	N1	A period in which the economy deteriorates significantly, characterized by inflation, unemployment, stock market drop, and lack of investment (a reference to the 2008 crisis).
Education	B, A, N1, N2, C	Acquiring knowledge in various fields. B: Education was judged to have an impact on the entire food waste system. N1: Formal education/large-scale education. N2: Formal education (a reference to nutrition, ecology, critical thinking, and political science).
Egocentrism	N2	Tendency to focus on the well-being of oneself, regardless of the negative consequences that their behavior might have on the rest of the society/natural environment.
Environmental/food waste awareness	B, A, N1, N2, C	Knowledge/interest regarding the natural environment, its vulnerabilities, and how it must be protected. Knowledge of the extent, impact, and solutions to food waste.
Fast pace of living (unexpected events)	B	Day-to-day disruptions of the everyday routine, unanticipated ‘change of plans’. It can have a negative impact on how individuals/households plan their meals.
Fear	N1	The emotional response that individuals/communities have in times of crisis, which has an impact on their behavior (short- and long-term).
Food collection and redistribution capacity	N1	The ability of a society to distribute its food resources efficiently, reduce food waste, and ensure the satisfaction of the needs of multiple categories of actors.
Food preparation	C	How raw ingredients are cooked, both for households and restaurants
Food quality	C	It was perceived as a way to differentiate between various items.
Food safety	C	It was linked to quality, with an impact on how consumers perceive expiration dates.
Geo-political crises	N1	Political instability/conflicts/wars (a reference to the war in Ukraine/communism in Romania).
Governmental intervention	A, N1, N2	Measures taken by the public administration to solve a specific issue (through campaigns, legislation, and strategies for allocation of resources).
Historical hardships	N1	Past negative experiences of a community that impact their current/future behavior (a reference to communism in Romania and the shortage of products during that time).
Holiday celebration	B	Celebrating various events with family/friends, preparing festive meals on those occasions.
HORECA/retails overstock	B	The tendency of businesses to purchase large quantities of food, higher than the consumer demand.
HORECA overproduction	B	The tendency of organizations to cook excessive amounts of dishes, higher than the consumer demand.
Household overproduction	B, C	The tendency of individuals/households to cook more food than needed.
Human error	N1	Reference to human error associated with labeling/packaging/transporting food products, which will make them improper for retail sale.
Human resource capacity	N1, C	N1: reference to the characteristics of the human resource (size, competencies), which impact their efficiency in a particular domain. C: capacity of farmers to protect their crops from animal damage.
Humans being connected to their food	N2	The capacity of humans to understand the real value of food (financial, natural resources used for production, human resource, impact on our health).
Individual/household overbuying/overstocking	B, N1, C	The tendency of individuals to purchase more food products than they need for consumption. N1: Usually in response to previous negative experiences with food, periods of hardship, or food scarcity.
Influence of religion	N1	The ability of religious organizations to influence the values/behaviors that a specific society has during a determined period.
influencers	B	Public figures with extensive outreach. This can refer to both people who raise awareness regarding food waste/environmentalism, but also people who promote consumerism.
infrastructure	C	Reference to composting collection points and facilities or to the infrastructure needed to protect crops from wild animals.
Interest in reducing food waste	B	The desire/willingness to take measures against food waste.
Investment/subsidies for local/eco products	N2	The allocation of material resources and know-how to local, small-scale farmers in an efficient manner.
Legislation	N1	The process of making or enacting laws (a reference to interventions of the public authorities in solving an issue).
Local resilience	N2	The ability of local communities to adapt when fluctuations in the global economy occur, the ability of a community to rely on its resources.
Market offer	A	The variety of food products that can be found on the market (a reference to precooked meals).
Marketing	B	The process of promoting certain food products, can refer to both the promotion of ‘ugly’ or ‘perfect’ products.
Mass-production	N1	The efficient and standardized industrial food system, which produces large quantities of less expensive food.
Meal/shopping planning	B	Anticipating the food needs of an individual/household, adapt the purchased amount.
Modern family dynamic	A	The dissolution of gender stereotypes regarding men’s and women’s roles in a household. The increased focus of women on developing professional careers.
Modern lifestyle	A, C	Characterized by the use of technology, post-material values, and increased standard of living.
Monitoring data	A	Reference to the collection and interpretation of food waste data (quantity wasted, categories of products, and trends).
‘Online food’	A	The possibility of buying food online, without going out of the house and seeing/picking the products yourself.
Overemphasizing appearance/abundance	B	The strategy of food providers to attract customers by presenting never ending full displays of good-looking food.
Processed/precooked food over home cooking	A	The tendency of modern society to opt for precooked meals and cook less at home.
Producer overproduction/waste	A, N1	The excessive production of food that surpasses demand.
Proper storage	C	Adapting storage conditions according to the specificity of a particular food product (e.g., refrigeration for perishable goods).
Prototype food products	N1	Food products that a company develops to expand its offer and gain a competitive advantage, can be disliked by consumers and not purchased.
Public campaign	A	A method through which public authorities can increase interest/awareness in a particular topic.
Resistance to change	N1	The tendency of individuals/communities to uphold past established values, norms, and behaviors, coupled with a negative reaction to novelty.
Retailer overstock	N1	Purchasing excessive amounts of food from producers, which is not matched by consumer demand.
Income	B, C	An increase in the financial means available to individuals/households, impacts the possibility of purchasing food.
Romanian social/cultural factors	B	Cultural and historical characteristics that define how Romanians relate to food (e.g., purchasing and preparing large quantities of food, the anxiety of being out of food).
Schools	A	Institution to deliver formal/large-scale education.
Self-respect	N2	Treating the physical and mental state with care (a reference to a healthy lifestyle and proper nutrition).
Social media	B	An online platform on which various people/ organizations can disseminate information, with a large reach. It can have a positive and negative impact on reducing food waste, depending on the type of content.
Solution/innovation	N1	The development of new strategies to solve a specific problem.
Strategies to deal with excess food	B	Process of anticipating food waste and preparing solutions.
Technological-based planning tools	B	Software, apps, and devices, which can help individuals and retailers keep track of their food resources, food needs, and characteristics of the items (viability, method of cooking, and proper pairings).
Time management	A, C	Increased interest in having more free time and spending it on pleasant activities.
Transition from materialism to post-materialism	A	Change in individual/social values when a level of economic prosperity is achieved. Focus on increasing life quality and self-development.
Transparency (retail, public authorities)	N2	Degree of openness, providing the public with information about the activity, performance, objectives.
Transportation	C	Food transportation conditions can have an impact on food quality.
‘Ugly food’ rejection	B, N1, C	The tendency of consumers to avoid purchasing/consuming food products which do not comply with strict cosmetic requirements, impacts the behavior of retailers and producers.
Understanding of the food system (end-to-end)	N2	Awareness and knowledge of the role of the food chain actors (from production, transportation, retail, to consumers) and their contribution to food waste production.
The perceived risk of food waste	A	Tendency to regard biodegradable waste as less problematic than other types of waste, such as plastic. Mainly related to the amount of time that it takes for each type of waste to decompose.
Waste management	N2	The large-scale problem of our modern society, in which a series of actors are involved (citizens, public authorities, companies).

^1^ “A”—local public administration, “B”— business sector on food waste reduction, “C”— consumers, “N1”—NGO representative 1, “N2”—NGO representative 2.

**Table 2:** Reinforcing and balancing loops from the food waste loop diagram.

Reinforcing Loops		Balancing Loops	
R1	Environmental/food waste awareness, government intervention, understanding the food system (end-to-end), education	B1	Education, income, individual/household overbuying/overstocking
R2	Governmental intervention, education	B2	Education, understanding the food system (end-to-end), governmental intervention, environmental/food waste awareness, individual/household overbuying/overstocking
R3	Governmental intervention, understanding the food system (end-to-end), education	B3	Education, understanding the food system (end-to-end), food waste
R4	Consumerism, retailer overstock, producer overproduction/waste	B4	Education, understanding the food system (end-to-end), Food waste, distinction between ‘best before’ vs. ‘sell-by dates’
R5	Accessibility of food, consumerism, ‘ugly food rejection’, food waste	B5	Environmental/food waste awareness, ‘ugly food’ rejection, producer overproduction/waste
R6	Education, income, household overproduction/large portions, modern lifestyle		

## Data Availability

Data are available upon request from the first author.

## Appendix A

All the loop diagrams elaborated during the group modeling sessions can be observed in this section. They are illustrated in the chronological order in which the workshops took place.
